# Khat Chewing among Students of Higher Education in Jazan Region, Saudi Arabia: Prevalence, Pattern, and Related Factors

**DOI:** 10.1155/2013/487232

**Published:** 2013-06-26

**Authors:** Rashad Mohammed Alsanosy, Mohamed Salih Mahfouz, Abdelrahim Mutwakel Gaffar

**Affiliations:** ^1^Substance Abuse Research Center (SARC), Jazan University and the Department of Family and Community Medicine, Faculty of Medicine, Jazan University, P.O. Box 114, Jazan, Saudi Arabia; ^2^Family and Community Medicine Department, Faculty of Medicine, Jazan University, P.O. Box 2531, Jazan 45142, Saudi Arabia

## Abstract

*Objectives*. (1) To estimate the prevalence and behavioral patterns of Khat chewing and (2) to investigate factors that influenced the pattern of Khat use among undergraduate students in different higher education institutions in Jazan region, Saudi Arabia. *Materials and Methods*. A cross-sectional study using a pretested structured self-administered quantitative questionnaire was used to collect data. SPSS version 17 software program was used for data analysis. *Results*. The overall current Khat chewing prevalence among higher education students was found to be 23.1%, significantly higher among males at 38.5% than among females at 2.1% *(P* < 0.001). Lifetime Khat chewer students were 24.8% for males at 40.5%, significantly higher compared with females at 3.7% *(P* < 0.001). Univariate analysis revealed that the gender of student, smoking status of student, a friend's smoking, and Khat chewing were associated with a significant high risk of Khat chewing (*P* < 0.001  * for all*). *Conclusions*. The use of Khat trend is increasing among higher education students in Jazan region. A multilevel, value based, comprehensive, and strategic long-term intervention plan is needed. The comprehensive plan may include social interventions geared by creating recreations alternatives and opportunities for youth and a critical review for current authorities' interventions and services.

## 1. Introduction

Health is defined as a state of complete physical, mental, and social well-being and not merely absence of disease or infirmity [1]. The definition is applied to physical, mental, psychological, social, and spiritual aspects of the individual person. During the past forty years, there has been a growing concern regarding the risk factors associated with youth physical health and mental well-being [2]. Substance abuse is among the most risk-taking behaviors committed by the youth that result in serious life-threatening consequences such as incarceration, disease, and death [3, 4].

Khat chewing is a common habit among all segments of Jazan population. Khat chewing produces psychostimulation effect in the form of euphoria and excitement because of cathinone contents [5–7]. It is well documented that Khat has many severe public health and social problems [8–14]. In addition to the health problems associated with Khat use, Khat chewers spend considerable time in this habit; this time wasting affects the social and economic development of the country. Diversion of family income for purchase of Khat results in neglecting the family needs leading to family conflicts and discords. Many studies have demonstrated that there is a clear association between heavy consumption of Khat and psychosis [15–21].

Adolescence means the period of transition from childhood to adulthood during which they experience enormous physical, psychological, and sexual changes. The economic, social, cultural, and political environments in which they live either directly or indirectly influence these changes. This critical stage of the life cycle which adolescents go through needs information and services carefully tailored to meet their needs [6].

Few studies have been conducted in Jazan region focusing on Khat chewing practice among secondary and university students [5–7]. University-wide surveys are needed to highlight Khat chewing patterns and different risk factors that may contribute to adoption of the habit among Saudi youngsters, comparing students of different sexes, majors, and school education years. Thus, the objectives of this study were to estimate the prevalence and behavioural patterns of Khat use among undergraduate students at higher education institutions of Jazan region, Saudi Arabia, during the academic year 2011/2012 and to investigate different associated factors that contribute to Khat chewing habit among university students.

## 2. Material and Methods

### 2.1. Design and Study Participants

A cross-sectional study targeted students in the three higher education institutions of Jazan region; they are Jazan university, the Technical College, and the Academic Health College (Private). Jazan University is a leading institution in the region with 26 faculties enrolling more than 50,000 students. Jazan region is one of the thirteen regions of the Kingdom of Saudi Arabia. It is located on the tropical Red Sea coast in southwestern Saudi Arabia. Jazan covers an area of 11,671 square kilometers, including some 5,000 villages and towns. Attached to it are 100 islands, including the largest island of Farasan. Jazan region runs along the Red Sea coast for almost 200 miles (300 km). It is a highly populated state with a total population of 1.5 million.

### 2.2. Sampling

A representative sample of 4,100 students was calculated, based on an estimated prevalence of Khat chewing use in Jazan region of 25% [6]. The sample stratified first according to the three institutions and then according to colleges. Clusters of classes were taken from each stratum randomly. For execution of the sampling plan, the sampling frames for selection of study participants were prepared in consultation with the deanship of student affairs and respective faculties to obtain details of classes and number of students in each faculty. Probability proportional to size sampling (PPS) was used to determine the number of students in the different faculties. 

### 2.3. Data Collection

A questionnaire was developed by reviewing the relevant literature and previously used standardized instruments and protocols [6, 7]. It contained 75 questions, most of which were closed-ended with precoded responses. The questions were divided into four sections: (i) sociodemographic backgrounds; (ii) Khat chewing habit; (iii) smoking and other substances; and (iv) attitudes towards Khat and smoking. The questionnaire was pretested in two selected colleges, and appropriate revisions were made before being used for actual data collection. 

The questionnaires were distributed and collected by class student's leaders under supervision of reaching assistants. The anonymity of subjects was emphasized and confidentiality strictly maintained on all collected study questionnaires. The operational definitions used were (a) nonuser: a person who has never used Khat in any form, (b) lifetime/ever chewer: an individual is considered an ever chewer even if he/she had chewed only once in his/her lifetime and (c) current user: individuals who were chewing Khat within 30 days preceding the study.

### 2.4. Data Quality Control and Analysis

To ensure data collection quality, fieldwork supervisor reviews the submitted questionnaire daily, and any errors or inconsistency is reviewed immediately. The data entry stage was started immediately after data collection was finished. The data entry took place at Jazan Substance Abuse Research Centre (SARC) under the supervision of data analysis specialist. The data were entered into Epi Info version 3.5.3 and transferred to SPSS version 17 (SPSS Inc., Chicago, IL, USA). Double data entry was conducted to ensure high quality of data. Data analyze involved descriptive statistics as well as inferential statistics. Descriptive statistics involved simple tabulation, frequencies, and proportion for categorical variables including cross-tabulations. Differences in proportions were compared for significance using chi-square test, with significance level set at *P* < 0.05. Also, multivariate logistic regression analyses were used to identify factors associated with Khat use by controlling for the effect of potential confounding variables. Khat chewing status was set as a dependent variable, while the following factors were included in the model as independent variables: gender, faculty type, class, smoking status, friend's smoking and Khat chewing status, and father's and brother's Khat chewing status. Adjusted odds ratios (ORs) and their 95% confidence intervals were reported. Hosmer-Lemeshow statistics was used to evaluate the goodness of the fit of the model.

### 2.5. Ethical Clearance

Ethical clearance was obtained from Jazan University Ethical Committee. Authorization was granted from his Excellency Vice-Chancellor of Jazan University and the deans of the respective colleges and faculties. Consent was granted from participating students during distribution of the questionnaire, and students were informed that the information collected will be kept anonymous and participation is totally voluntary. 

## 3. Results

The overall response rate for distributed questionnaires was 91.80% (3764 from the target of 4100 students), but not all questions were attempted equally by all participants. The mean, median, and mode of students ages were 20.8, 21.0, and 20.0 years, respectively, with (SD = 1.5), which indicates a normal distribution of the participants ages. As shown in Table 1, 57.5% of students were males compared with 42.5.5% females. Most sampled students were single (86.5%). One-quarter of the students were from health-related colleges (24.7%), while students from the other two groups Arts and Humanities and other science colleges were 36.7% and 38.6%, respectively, which represent the actual student distribution in higher education institutions in Jazan. Health-related colleges includ medicine, dentistry, pharmacy and applied medical sciences; other scientific colleges involved science, scientific specialization of education faculties, and computer science. The third category Arts and Humanities included arts, education, language and translation, administrative sciences, and Sharia and law. With regard to the area of residence, most students (58.8%) were from urban background and the remaining (41.2%) from rural areas (Table 1). The parental education of the respondents revealed that 38.8% of the mothers and 14.8% of the fathers were reported to be illiterate, with only 11.6% of the mothers and 27.1% of the fathers with university degree or above (Table 1).

Table 2 provides the lifetime and current prevalence of Khat chewing among study participants; the overall current Khat chewing prevalence among students was found to be 23.1% (95% CI: 21.7–24.4), significantly higher among males at 38.5% (95% CI: 36.5–40.5) than among females 2.1% (95% CI: 1.6–2.9) (*P* < 0.001). The same indicator was found to be significantly lower for urban students at 22.2% compared with 28.6% for rural students (*P* < 0.001). The current Khat chewing prevalence among scientific colleges, health-related faculties, and Arts and Humanities, 29.7%, 23.5%, and 15.8% respectively. 

Lifetime Khat chewers students were 24.8% (95% CI: 23.4–26.2), for males at 40.5% (95% CI: 38.4–42.5) also significantly higher compared with females 3.7% (95% CI: 4.39–6.53) (*P* < 0.001). The lifetime prevalence of Khat chewing rate for rural students was 26.5%, significantly different from urban students 20.6% (*P* < 0.05). The ever Khat chewing prevalence was found to be 23.5% in health related colleges, compared with 15.8% and 23.1% for Arts and Humanities and other science colleges.

Table 3 shows patterns of Khat chewing among both male and female students. It is clear from the table that the majority of students chew Khat in their houses followed by friend's house with significant difference between male and female responses (*P* < 0.001); more than 50% of females chew Khat in their houses compared with 36.9% of boys. Although Khat is banned by the government, the majority of participants (89.1%) argued that it is easy (38.9%) or to some extent easy (50.2%) to obtain Khat; no significant difference was observed between males and females responses (*P* > 0.05). Almost 53.7% of students buy Khat directly from sellers; 54.8% of boys buy it, while 29.4% of girls said that they take some from another person (*P* < 0.001). Students did not show different responses regarding age at first time of Khat chewing; the majority (63.5%) reported that they started chewing Khat after the age of 15 years. Regarding the frequency of Khat chewing, 78.3% said that they chew Khat in social occasions only, while 29.3% of the girls chew Khat at a daily bases, and 36.6% of the boys chew Khat at weekends (*P* > 0.001). When students were asked about timing of Khat chewing, 78.3% of them reported that they chew Khat at night with significant difference between boys and girls (*P* < 0.001) as 23.8% of girls prefer to chew Khat at noon; only 8.1% of boys chew Khat at the same time. Further, there is a significant difference between male and female responses regarding with whom do they chew Khat, time spent in Khat chewing session, and motives for Khat chewing *P* value = 0.000, 0.023, and 0.000, respectively. Almost 62.3% of students spend between (5 and 9) hours in Khat session, and 70.1% of them chew Khat with their friends, while approximately 50% chew Khat when they feel they need to change their mood.

The results of the univariate and multivariate logistic regression analyses for potential risk factors of Khat chewing are shown in Table 4. Univariate analysis revealed that gender of student, smoking status of student, friend's smoking and Khat chewing, and father's and brother's Khat chewing status were associated with a significant high risk of Khat chewing (*P* < 0.001  *for all*), students' smoking status (OR = 20.69, *P* < 0.001), gender (OR = 17.7,  *P* < 0.001), friend using Khat (OR = 17.13, *P* < 0.001), friend using tobacco (OR = 7.11, *P* < 0.001), brother using Khat (OR = 2.56, *P* < 0.001), and father using Khat (OR = 1.77,  *P* < 0.001). In the multivariate logistic regression analysis, students' smoking status (OR = 18.25, *P* < 0.001); gender (OR = 10.10, *P* < 0.001); friend using Khat (OR = 4.52,*P* < 0.001); brother using Khat (OR = 2.65, *P* < 0.001); father using Khat (OR = 1.68, *P* < 0.05) remained as significant and independent predictors for Khat chewing among higher education students of Jazan region.

## 4. Discussion

Six years ago, the prevalence of current Khat chewing among college students in Jazan was 15.2% among males [5, 6] in spite of the efforts done by government and NGOs, the present study showed that the overall prevalence reaches 23.1% (38.5% for males and 2.1% for females). The prevalence of Khat chewing among higher education students is less than that observed among general population in Jazan, Yemen, Ethiopia, and Somalia [7, 17, 22, 23]. Among Aden University medical students in Yemen, 54% chew Khat [22]. Similar prevalence of rate of current use of khat (22.3%) was reported in Ethiopia among medical and paramedical students in a college in northwestern Ethiopia [23] and among 20.4% among students in southwestern Uganda [24]. Lifetime Khat chewing prevalence in the current study was 24.8% (males 40.5%, females 3.7%) which is similar to that reported in Jazan previously [6] and lower than that reported in Ethiopia [23]. The use of Khat (current and lifetime) was significantly higher among males and rural residents; similar results about the residents were reported in Jazan previously and Ethiopia [6, 25]. 

Although Khat trading is illegal in Saudi Arabia, and since 2006 a new more strict legal system was implemented, it seems to have no effect, because there is an observed rising trend in Khat use among higher education students, and it can be contributed to the availability of Khat and easiness to get it. The majority of the participants—almost 90%—stated that it is easy or to some extent easy to get Khat. Jazan region is bordering Yemen and across the Red Sea in Ethiopia, Somalia, and Kenya where Khat production and trading is legal [26], and smuggling is difficult to control. During the period 2009–2011 the “Al Hothian war” north of Yemen affected the “accessibility” and “increased the cost,” as believed by many people in Jazan. 

Also Faifa mountain area in Jazan is famous for Khat production. The government prohibits the expansion of khat cultivation in Faifa mountain and established Faifa Development Authority since 1978 under the supervision of the Ministry of Interior to control Khat cultivation and support farmers to replace Khat with alternative crops, such as fruit and coffee trees [6]. The progression of such intervention need to be assessed because Khat is still cultivated in Faifa, and strengthening of mechanisms for law enforcement is needed to minimize the availability of Khat in the community. 

The pattern of Khat use among higher education students is not restricted by social regulation mechanisms, and even, it is a social norm. It is an acceptable and socialization practice; only 8% of users chew Khat alone, while the rest use Khat with their family members, relatives, and friends [27]. Female use pattern is significantly different from male users regarding the partners (Table 3), Khat source, place, chewing time, and duration. Females tend to use Khat more with family and relatives, get it from another person, use it at home at noon, and chew for less than five hours, while males use khat more frequently with friends, purchase it, use it at friends' houses and public places at night, and chew for more than five hours. Those who spend less than 5 hours chewing Khat were 25.2%, while it was 48.2% in 2006 [5]. Understanding these different patterns of Khat use is important when planning community intervention programs to reach users.

Alcohol and drugs use in different cultural backgrounds share a common hypothetical causal mechanism. “Killing time” is the similar function of drugs and alcohol use across groups which involve countering the psychosocial distress and uncertainties about the future as well as past trauma [28]. People use Khat for similar reasons: when they need to change their mood (49%) and feel stressed (8.8%). Khat is used usually for social recreation; motor vehicle drivers and truck drivers in Ethiopia chew khat during long distance driving to keep awake. During examination periods, a significant portion of students chew khat to be alert. To reduce physical fatigue is one of the functions that craftsmen and farmers use khat for, and traditional healers use it to heal ailments [29]. Generally, many social determinants interact with khat use. The monotony of life/work which leads to behaviors for “killing time” along with heavy academic load forces students to use this stimulant as reported in other countries. 

In a study among Yemeni Khat consumers, they argue that khat improves relationships in Yemeni society, is the best way to spend one's time, leads to relief of social and mental stress, and improves sexual experience during intercourse [30]. The available alternatives for youth to spend their time are limited in the region. Thus, options for entertainment, voluntary work, open spaces like parks and playgrounds, clubs, sport, and recreational activities are highly needed. 

The most significant risk factors identified in this study (Table 4) were students' smoking status, male gender, having friend using Khat, brother using Khat, and father using Khat and are significant and independent predictors for Khat chewing among higher education students of Jazan region. 

Similar factors are also reported as risk factors for tobacco use like friends, brothers due to peer pressure, and the influence of friends. 

## 5. Conclusions and Recommendations

The use of Khat trend is increasing among higher education students in Jazan region, and it reflects the social and legal status of Khat in the community. Although Khat is illegal in Saudi Arabia, it is both socially acceptable and easily available and accessible by the majority of the population. Prevention and control programs need to use both high risk groups and general population interventions approaches. Law enforcement alone will not be sufficient to control and prevent Khat chewing in Jazan community. A Multilevel, value based, comprehensive, and strategic long-term intervention plan is needed for more effective tools for preventive intervention and legal systems. The comprehensive plan may include, social interventions to change the community norms regarding Khat use which is crucial, geared by creating recreations alternatives and opportunities for youth, assessment and review of Faifa Development authority project for replacing Khat cultivation, reinforcement of law, and services programs for those at risk or need help to quit Khat use.

## 6. Study Limitations

Although Khat use is socially acceptable in Jazan region, we could not collect data about some associated behaviors like alcohol and sexual behaviors. Alcohol use is banned by law in Saudi Arabia and not tolerated socially and from Islamic religious point of view. The previously mentioned fact is applicable about sexual behaviors, and sometimes it is considered humiliating to ask such questions. The nonresponse rate is one anticipated issue. We controled for this by calculating the sample to overcome the expected response rate. 

## Figures and Tables

**Table 1:** Some selected background characteristics of the study population.

Characteristics	*N*	%
Gender (*n* = 3764)		
Male	2165	57.5
Female	1599	42.5
Age groups (*n* = 3217)		
Less than 20 years	614	16.3
20-21	1606	42.7
22-23	830	22.1
24 and above	167	4.4
Colleges (*n* = 3764)		
Health related	931	24.7
Arts and humanities	1380	36.7
Other scientific	1453	38.6
Mode of living		
Rural	1549	41.2
Urban	2215	58.8
Marital status (*n* = 3764)		
Married	341	9.1
Single	3255	86.5
Divorced	44	1.2
Widowed	5	0.1
Father's education (*n* = 3764)		
Illiterate	557	14.8
Primary	981	26.1
Intermediate	595	15.8
Secondary	521	13.8
University and above	1021	27.1
Mother's education (*n* = 3764)		
Illiterate	1459	38.8
Primary	1068	28.4
Intermediate	419	11.1
Secondary	306	8.1
University and above	437	11.6

**Table 2:** Prevalence of Khat chewing among study participants.

Category	Ever Khat chewers		Current Khat chewers	
	*N* (%)	95% CI	*N* (%)	95% CI
*Gender *				
Male	876 (40.5)**	38.4–42.5	834 (38.5)*	36.5–40.5
Female	59 (3.7)	2.9–4.9	34 (2.1)	1.6–2.9
*College Type *				
Health related	221 (23.7)	21.1–26.5	219 (23.5)	20.9–26.3
Arts and humanities	248 (18.0)	16.0–20.0	218 (15.8)	18.9–17.8
Other scientific	466 (32.1)	29.7–34.5	431 (29.7)	27.3–32.0
*Mode of living *				
Rural	443 (28.6)*	26.4–30.9	441 (26.5)*	24.3–28.8
Urban	492 (22.2)	20.5–23.9	457 (20.6)	18.9–22.3

Total	935 (24.8)	23.4–26.2	868 (23.1)	21.7–24.4

^∗^Significant difference (*P* value < 0.05), **significant difference (*P* value < 0.01).

**Table 3:** Pattern of Khat chewing among study participants according to gender.

Characteristics	Male *N* (%)	Female *N* (%)	Total *N* (%)	*P* value
Place of Khat chewing (*n* = 857)				0.000
In my house	299 (36.9)	25 (53.2)	324 (37.8)	
In my friend's house	215 (26.5)	10 (21.3)	225 (26.3)	
In school	7 (0.9)	5 (10.6)	12 (1.4)	
In public places	165 (20.4)	2 (4.3)	167 (19.5)	
In occasions	25 (3.1)	0 (0.0)	25 (2.9)	
Other place	99 (12.2)	5 (10.6)	104 (12.1)	
Do you think that it is easy to obtain Khat? (*n* = 862)				0.378
Yes	313 (38.5)	22 (44.9)	335 (38.9)	
Yes to some extent	413 (50.8)	20 (40.8)	433 (50.2)	
No	87 (10.7)	7 (14.3)	94 (10.9)	
Khat source (*n* = 738)				0.000
From sellers	386 (54.8)	10 (29.4)	396 (53.7)	
I give money to another person to buy it	67 (9.5)	4 (11.8)	71 (9.6)	
I take some Khat from another person	117 (16.6)	10 (29.4)	127 (17.2)	
Elder person gives me Khat	55 (7.8)	5 (14.7)	60 (8.1)	
Other	799 (11.2)	5 (14.7)	84 (11.4)	
Age at first time to chew Khat (*n* = 868)				0.653
Less than 10 years	67 (8.0)	4 (11.8)	71 (8.2)	
10–14 years	238 (28.5)	8 (23.5)	246 (28.3)	
More than 15 years	529 (63.4)	22 (64.7)	551 (63.5)	
Khat chewing time (*n* = 847)				0.000
At noon	65 (8.1)	10 (23.8)	75 (8.9)	
After noon	66 (8.2)	4 (9.5)	70 (8.3)	
At evening	38 (4.7)	1 (2.4)	39 (4.6)	
At night	636 (79.0)	27 (64.3)	663 (78.3)	
Frequency of Khat chewing (*n* = 805)				0.005
Daily	57 (7.5)	12 (29.3)	69 (8.6)	
Most of the weekdays	86 (11.3)	8 (19.5)	94 (11.7)	
Weekends only	280 (36.6)	9 (22.0)	289 (35.9)	
In occasions	341 (44.6)	12 (29.3)	353 (43.9)	
Time spent in Khat session (*n* = 822)				0.000
Less than 5 hours	190 (24.3)	17 (43.6)	207 (25.2)	
5–9 hours	500 (63.9)	12 (30.8)	512 (62.3)	
More than 10 hours	93 (11.9)	10 (25.6)	103 (12.5)	
With whom do you chew Khat (*n* = 849)				0.023
Family	41 (5.1)	5 (10.9)	46 (5.4)	
Relatives	131 (16.3)	8 (17.4)	139 (16.4)	
Friends	570 (71.0)	25 (54.3)	595 (70.1)	
Alone	61 (7.6)	8 (17.4)	69 (8.1)	
Reasons of Khat chewing (*n* = 817)				0.000
When feeling their stressed	61 (7.9)	11 (25.0)	72 (8.8)	
Anxiety	25 (3.2)	2 (4.5)	27 (3.3)	
Need to change mood	375 (48.5)	25 (56.8)	400 (49.0)	
Other reasons	312 (40.4)	6 (13.6)	318 (38.9)	

**Table 4:** Univariate and multivariate logistic regression analyses for Khat chewing related factors among study participants.

Category	Univariate			Multivariate^#@^		
	OR	95% C.I.	Sig.	OR	95% C.I.	Sig.
Gender						
Female (Ref.)						
Male	17.7	13.4–23.3	0.000	10.11	4.88–17.38	0.000
College type (Ref.)						
Other scientific						
Health-related	0.70	0.57–0.86	0.001	1.33	0.87–2.03	0.184
Arts and humanities	1.52	1.26–1.82	0.000	1.67	1.13–2.47	0.011
Academic year						
First						
Second	1.61	1.28–2.02	0.000	1.01	0.68–1.50	0.965
Third	1.55	1.23–1.96	0.000	1.024	0.67–1.55	0.911
Fourth	1.42	0.98–2.06	0.065	1.44	0.76–2.74	0.268
Smoking status						
No (ref.)						
Yes	20.69	16.96–25.25	0.000	18.25	12.95–25.72	0.000
Friend's smoking status						
No (ref.)						
Yes	7.11	5.90–8.56	0.000	1.243	0.84–1.83	0.272
Friend's using Khat status						
No (ref.)						
Yes	17.13	13.39–21.92	0.000	4.43	2.78–7.37	0.000
Father using Khat						
No (ref.)						
Yes	1.77	(1.51–2.07)	0.000	1.68	1.22–2.31	0.001
Brother using Khat						
No (ref.)						
Yes	2.56	(2.18–2.99)	0.000	2.65	1.93–3.65	0.000
Feeling Stressed						
No (ref.)						
Yes	1.21	(1.03–1.42)	0.020	0.97	0.64–1.46	0.87
Feeling depressed						
No (ref.)						
Yes	1.07	(0.90–1.26)	0.431	1.38	0.91–2.08	0.125

^@^Hosmer-Lemeshow goodness of fit test *χ* ^2^ = 3.181, *P* = 0.923; ^#^adjusted for other variables in the table.
